# Use of methamphetamine and alcohol among people with opioid use disorder and HIV in Vietnam: a qualitative study

**DOI:** 10.1186/s12889-021-11783-9

**Published:** 2021-09-22

**Authors:** Andrew Edsall, Kim A. Hoffman, Dinh Thanh Thuy, Pham Phuong Mai, Nguyen Thu Hang, Tong Thi Khuyen, Nguyen Thu Trang, Lynn E. Kunkel, Le Minh Giang, P. Todd Korthuis

**Affiliations:** 1 Oregon Health & Science University School of Medicine, 3181 SW Sam Jackson Park Rd, Portland, OR 97239 USA; 2 Oregon Health & Science University - Portland State University School of Public Health, 840 SW Gaines St, Room 230, Portland, OR 97239 USA; 3 Hanoi Medical University, 1 Tôn Thất Tùng, Kim Liên, Đống Đa, Hà Nội, 116001 Vietnam; 4 Department of Medicine, Section of Addiction Medicine, Oregon Health & Science University, 3181 SW Sam Jackson Park Rd, Portland, OR 97239 USA

**Keywords:** Methamphetamine, Alcohol, Heroin, HIV, Vietnam, Medication for opioid use disorder

## Abstract

**Background:**

Heroin use continues to drive HIV transmission in Vietnam, but methamphetamine and alcohol use are growing rapidly and, as in other countries, polysubstance use is widespread. The objective of this study was to understand the interplay between heroin, methamphetamine, and alcohol use among people with opioid use disorder (OUD) and HIV in Vietnam.

**Methods:**

We conducted 44 in-depth, face-to-face qualitative interviews with people with OUD and HIV who participated in the BRAVO trial of buprenorphine versus methadone in five Vietnam HIV clinics. Interviews probed participants’ experiences of heroin, methamphetamine, and alcohol use and their interplay with HIV/OUD treatment. Interviews were professionally transcribed and analyzed using a thematic analysis approach.

**Results:**

Of 44 participants interviewed 42 were male, on average 38.8 years of age, with 30 reporting a history of methamphetamine use and 33 reporting a history of alcohol use. Several themes emerged: 1) Methamphetamine and alcohol were perceived to have lower addiction potential than heroin 2) Social settings were key facilitators of alcohol and methamphetamine use 3) Some participants, but not all, used methamphetamine to help quit heroin 4) Consuming alcohol blunted the effects of heroin, while paradoxically serving as a catalyst for heroin use 5) Use of methamphetamine was perceived by many participants to be incompatible with treatment for HIV.

**Conclusions:**

Participant experiences reflected a significant impact of polysubstance use on treatment of HIV and OUD. Patterns of polysubstance use are subject to common preconceptions of alcohol and methamphetamine as having a low addictive potential, and these substances are deeply enmeshed in the social life of many people with OUD in Vietnam. Interventions to address complex social norms and potential harms of polysubstance use are urgently needed as the population of people receiving medication for OUD (MOUD) increases in Vietnam and globally.

**Trial registration:**

BRAVO - NCT01936857, September 2013.

## Background

An estimated 57.8 million people worldwide use opioids, of whom 30.4 million use opiates such as heroin [1]. These estimates, based on recent epidemiologic studies, indicate that the global prevalence of opioid use disorder (OUD) is significantly higher than was previously thought. Injection drug use (IDU), of which opioid use accounts for an estimated 82.9%, represents a major risk factor for poor health outcomes [2]. At least 12% of people who inject drugs (PWID) were living with HIV in 2019, and PWID were 29 times more likely to acquire HIV than people who do not inject drugs [3]. Moreover, 52.3% of PWID have been exposed to Hepatitis C and an estimated 9% have chronic Hepatitis B [2].

Use of other substances is commonplace among people who primarily use opioids worldwide [4–7]. Alcohol is the most common secondary substance used among people who use drugs in Europe; in the United States 35% of people who use heroin meet criteria for alcohol use disorder [5, 7]. Alcohol use is also prevalent among patients receiving treatment for OUD: an estimated one-third of patients receiving medication for opioid use disorder (MOUD) have an alcohol use disorder [8, 9].

People with OUD commonly use methamphetamine, as well. Between 2011 and 2017 concomitant methamphetamine and opioid use in the United States doubled to 34.2%, and prevalence of methamphetamine and heroin co-use among those who inject is as high as 50% [10, 11]. The overall prevalence of amphetamine use in East and Southeast Asia is also above the global average, and in recent years the region has represented the fastest growing market for methamphetamine worldwide [1, 12]. Limited data suggests that methamphetamine is the most common stimulant used as secondary drug among people who primarily use opioids in Asia, with an estimated 33% of people with a history of heroin dependence in China reporting use of amphetamine-type stimulant (ATS) [13].

Polysubstance use among people on MOUD has been associated with lower rates of retention on these medications [14]. Concurrent use of either alcohol or methamphetamine specifically may decrease the effectiveness of OUD treatment among people who primarily use heroin [14]. In the United States, people who co-use methamphetamine and opioids are less likely to receive MOUD than people who use opioids only [15]. Alcohol abuse is associated with increased risk of return to opioid use for people treated with buprenorphine [16]. Among people with HIV, methamphetamine use has been associated with increased HIV risk behaviors and transmission [17, 18], higher HIV viral load, lower CD4+ T cell counts, HIV-related neuronal damage, and increased antiretroviral resistance [19–22]. Alcohol, too, is associated with behavioral and biological risk factors for HIV infection and progression including high-risk behaviors related to sex and injection drug use, lower adherence to antiretroviral therapy (ART), increased HIV viral load, and HIV-related central nervous system effects [23–27]. Alcohol is also known to potentiate the respiratory depressant effects of both opioids and opioid receptor agonists [28].

Patterns of polysubstance use among people who primarily use heroin may relate to underlying motivations for using other substances, but remain under-studied with regards to methamphetamine. Co-use of a different stimulant, cocaine, has been described as being either simultaneous, to enhance the effects of heroin, or sequential, to mitigate undesired effects including withdrawal [29]. These rationales may help to explain different patterns of heroin and methamphetamine co-use, including co-injection and separate use [11]. A growing body of qualitative literature has described key reasons for methamphetamine use among people with OUD. These include a desire to attain a synergistic high, balance the two substances’ relative effects, or mitigate the risk of withdrawal or overdose [10, 30, 31]. Moreover, methamphetamine use has been described amongst people receiving MOUD in order to provide an alternative high to opioids and/or to mitigate sedating effects of MOUD [31, 32].

Vietnam is home to an estimated 189,000 PWID, of whom 52,200 received methadone maintenance therapy in 2020 [33, 34]. Injection drug use remains a main driver of HIV transmission in Vietnam, with PWID accounting for 36% of new HIV diagnoses in 2015 [35]. Between 2005 and 2019 coverage of ART in Vietnam expanded to an estimated 70% of people living with HIV, many of whom have a history of OUD [33, 36]. Recent evidence suggests that polysubstance use is common among patients receiving MOUD in Vietnam, consisting primarily of alcohol, tobacco, and ATS [37]. Estimates of the prevalence of methamphetamine use among patients receiving MOUD in Vietnam range from 24 to 51% [38, 39]. A study of PWID in Northern Vietnam showed a prevalence of daily alcohol consumption of between 20 and 30%, in line with global estimates [40]. Understanding polysubstance use, which may influence outcomes of MOUD and ART use, is therefore important for managing Vietnam’s burden of both OUD and HIV. The objective of this study was to add to the literature regarding how and why people with OUD use methamphetamine and alcohol, specifically within the context of a developing nation where use of ATS is increasingly prevalent. Results may hold implications for future efforts to address SUD and HIV in Vietnam, as well as throughout Southeast Asia and beyond.

## Methods

We conducted a qualitative study of participants enrolled in the “Buprenorphine to Improve HIV Care Engagement and Outcomes” (BRAVO - NCT01936857) study which compared substance use and HIV outcomes among people with HIV and OUD randomized to receive HIV clinic-based buprenorphine or referral to methadone maintenance therapy treatment strategies for OUD in six HIV clinics in Northern Vietnam. The BRAVO study did not exclude participants with other drug use, but did exclude participants with AST or ALT greater than five times normal. Qualitative baseline interviews were conducted shortly after participant enrollment with a subset of participants, purposively sampled to achieve approximate balance in assigned treatment groups (buprenorphine versus referral to methadone maintenance therapy) and to maximize diversity of insights based on continuation on MOUD, marriage status, and employment status. Selected participants were initially approached by a research assistant during clinic visits to receive MOUD, HIV care, or BRAVO study procedures. All participants were informed of the study aims during the recruitment phase and completed written informed consent. The aim of this qualitative study was to describe the perspectives of people living with OUD and HIV with regards to polysubstance use. Institutional review boards at Oregon Health & Science University (IRB00000471) and Hanoi Medical University (IRB00003121) approved the study.

Interviews were conducted by trained qualitative interviewers with at least a Masters-level education who were employed full-time as research staff during the BRAVO study. One of two interviewers (one female and one male, both of whom had previous experience conducting qualitative interviews) conducted each interview. No relationship between researchers and participants existed prior to study commencement. Interviews were conducted face-to-face, in Vietnamese, in a private clinic room. Interviews lasted between 30 and 60 min and were digitally recorded. Interview topics included: general/demographic information, substance use, and current/prior substance use treatment. Participants received 200,000 Vietnamese Dong (about $10 U.S.) for each interview. Field notes were recorded during each interview to supplement recordings. At the end of each interview, participants were provided with an opportunity to further clarify or amend earlier statements. Between December 30, 2015 and April 27, 2018, interviews of 44 participants were conducted.

Interview recordings were professionally transcribed in Vietnamese. Thematic analysis was employed using a semantic, inductive approach to identify themes related to methamphetamine and alcohol use, as well as other areas of interest. Three researchers developed a list of preliminary codes after reading interview transcripts in Vietnamese, with codes grouped into overarching themes. An inter-coder reliability process was conducted in which the Vietnamese transcripts were coded by two coders using Atlas.ti software, with discrepancies adjudicated by a third coder. Ten percent of all transcripts were double-coded to achieve an intercoder reliability rate of 85%. Codes related to alcohol and methamphetamine use were translated into English, along with select quotations. Major themes related to methamphetamine use and alcohol use were described, along with minor themes and significant deviations from major themes.

## Results

Participants were 95% male; 39% were employed, and 36% were married. Any history of prior or current methamphetamine use was reported by 68% of participants either on a written questionnaire or in response to interview questions, while any history of prior or current alcohol use was reported by 75% of participants (Table 1).

**Table 1:** Participant Characteristics

**Treatment***(n = 44)*	
HIV clinic-based buprenorphine	23
Referral for methadone maintenance therapy	21
**Demographic Characteristics**	
Mean age (SD)	38.8 (6.4)
Male Sex	42 (95%)
Employed	17 (39%)
Married	16 (36%)
History of methamphetamine use	30 (68%)
History of alcohol use	33 (75%)

Five major themes emerged regarding use of methamphetamine and alcohol among study participants:

### Theme 1: methamphetamine and alcohol were perceived to have lower addiction potential than heroin

Most participants with a history of methamphetamine or alcohol use did not perceive themselves to be addicted to either substance, which they explicitly contrasted with heroin. In drawing a clear distinction between heroin and methamphetamine or alcohol, participants tended to normalize their use of methamphetamine and alcohol, and discounted potential harms compared to their use of heroin.

*“Because ice [methamphetamine] is not as addictive as heroin, methamphetamine users do not depend on it as much as they do on heroin. Users don’t have to use it regularly, they maybe use it today, but in the next days, they can stop using it without any withdrawal and pain symptoms, as is felt with heroin deficiency.”*

*“This ice doesn’t produce withdrawal, unlike heroin. So that I can do it as I want, to feel better, to avoid thinking. I can cease to use it if I wish.”*

Similarly, participants tended to frame their alcohol use as being within their conscious control. In contrast to methamphetamine, however, participants describe use of alcohol as a normal part of life rather than as a substance used intermittently or on special occasions.

*“Drinking two to three bottles of beer every day is for refreshing... Refraining from alcohol is unnecessary because I am not addicted to alcohol. You have to be addicted to heroin, then you have to try to quit it, but why do you have to refrain from alcohol if you only drink one to two bottles every day.”*

### Theme 2: social settings were key facilitators of alcohol and methamphetamine use

Most participants who used methamphetamine reported doing so in social settings, especially among groups of friends. This was reported for both introduction to methamphetamine as well as continued use.

*“I tried it around 2016…2017 around the Tet holiday. I remember it was New Year’s Eve, some addicted friends invited me to use ice with them. It was the first time I ever tried it.”*

*“Yes, I do ice sometimes. It’s so common now. You can find it everywhere. It’s like water pipe. You offer others a few smoke. If I like it, I’d smoke more...”*

Participants reported not only social drinking, but social pressure to consume alcohol. Examples included alcohol use related to work and traditional patterns of alcohol use such as regularly drinking rice-based medicinal alcohol *(Rượu thuốc)* before meals. Particularly strong social pressure was experienced on ceremonial occasions such as weddings, death anniversaries, and Tet, when it could be socially unacceptable to refuse alcohol.

*“In general, alcohol brings nothing, but for work and when in contact with people I have to drink. I cannot refuse it.”*

Participants reported alcohol and methamphetamine use both inside and outside the home. Methamphetamine was used in the company of friends or wider social circles, while alcohol was additionally used around immediate family.

*“Every day, I drink one and a half liter of wine dividing for three meals at home. My wife and I both have friend gatherings once or twice a month. We will drink there then.”*

*“I do not drink much… I drink three to four times day. If I am out with some friends, I will drink or when I am with my family I also drink. It is kind of a habit. When it is time for a drink, and if I have not drunk it yet, then I will drink it at a mealtime.”*

*“… And another thing is now many drug users like to go to the discos and bars so they normally use an amphetamine type stimulant like methamphetamine…”.*

*“Sometimes, I went to my friend’s house and in here we used ice together. I did not consume it so frequently. When we gathered together for fun, we used ice...”*

### Theme 3: some participants, but not all, used methamphetamine to help quit heroin

Participants reported a range of experiences with methamphetamine and heroin use, across various stages of use or disuse of both substances. Several participants reported that using methamphetamine helped them to use less heroin, or that it provided an alternative way to experience a high without using heroin.

*“First, people who are taking Methadone stop using heroin and change to using ice because now heroin does not give them as much high as before they started taking Methadone, which weakens the effect of heroin. Therefore, seeing this people would not choose to use heroin, and they would try to find some other substance which brings them a different feeling, because Methadone does not seem to affect ice. [For Methadone patients] using ice brings the feeling as same as for people not using Methadone.”*

*“I just think I give up one substance I will use a different substance. Honestly, heroin was more harmful, so I quit it. I like ice, but I am not addicted to it. I have used it for a long time. I am still using it now… I do not feel high or anything. I think I am a man and if I give up all these, it will be so humdrum.”*

However, even while acknowledging that many of their peers used methamphetamine in this way, other participants reported that methamphetamine actually increased their desire to use heroin.

*“Many people say that [methamphetamine use can help with quitting heroin], you understand? However, personally I do not think like that… Because I used it and I knew. Using ice cannot [help] quit heroin because it only makes us want to use heroin more… For me, after I used ice, I wanted to find heroin. It is really like that.”*

### Theme 4: consuming alcohol blunted the effects of heroin, while paradoxically serving as a catalyst for heroin use

Many participants reported trying to avoid consuming alcohol with heroin, as alcohol blunted the effects of heroin. However, several participants paradoxically reported that consuming alcohol made them want to use heroin more.

*“When I used heroin, I did not want to drink alcohol because drinking alcohol dampened heroin.”*

*“…I do not dare to drink because every time I drink, I will end up using heroin. After drinking alcohol, I tend to lose control, think about heroin, and really want to use it.”*

### Theme 5: use of methamphetamine was perceived by many participants to be incompatible with treatment for HIV

Participants’ relationship with methamphetamine varied with treatment for HIV. Prior to participating in the BRAVO study, some recalled having missed outpatient HIV care appointments as a result of their methamphetamine use.

*“Yes, it [using methamphetamine] makes it easier for me to forget some things. Using ice leads me forget the appointments with the clinic. Then, the healthcare providers have to make a phone call to me for reminders.”*

*“Maybe now I think about it. At that time when I used it [methamphetamine], I did not think about it. However, now I quit it, I usually think back. Maybe sometime in the past I did not take the medication [ART] on time. When I still used it, I was into it so I could not remember if I took the medication or not, so I might take the medication late or I might forget taking it.”*

## Discussion

Polysubstance use in Vietnam is best understood within the context of overarching substance use trends, as well as policy changes related to treatment for OUD and HIV. Since the passage of the Law on HIV/AIDS Prevention and Control in 2006, the Government of Vietnam has facilitated treatment implementation aimed at controlling the spread of HIV, including provision of MOUD to PWID and scale-up of ART [41]. This has given rise to a new and growing population of people living with HIV who have a history of OUD, many of whom are receiving long-term MOUD in addition to ART. During this same period, use of methamphetamine has increased significantly in Vietnam [42]. While heroin is used by 68% of the total population of people who use drugs, during the first 6 months of 2018 more than 60% of *newly identified* people who use drugs in Vietnam used methamphetamine, while fewer than 40% used heroin [42]. Per-capita alcohol consumption has also increased in Vietnam over the past 15 years, doubling among men and growing six-fold among women [43]. Multiple studies suggest that binge drinking increased during this time, with current estimates of the prevalence of binge drinking or hazardous alcohol use ranging from 31.7 to 52.9% among males [43, 44]. Against the backdrop of a growing number of people receiving long-term MOUD and ART in Vietnam, this increasing prevalence of methamphetamine and alcohol use has the potential to adversely affect treatment of OUD and HIV in the country.

Our qualitative study population was made up almost entirely of male participants, reflecting not only the composition of the overall BRAVO study population but also the demographic makeup of people receiving treatment for MOUD in Vietnam [34]. Substance use is widely believed to be a predominantly male phenomenon in Vietnam, perhaps due to traditional gender roles in the culture of Vietnam. However, little data exists to substantiate this belief, and it is possible that current treatment efforts are failing to reach significant numbers of females with SUD in Vietnam [34].

Most participants in our study felt unambiguously that their use of alcohol and methamphetamine did not constitute addiction. This may be related to broader societal views of these substances, although little research has been conducted into public attitudes towards alcohol and methamphetamine in Vietnam. In the case of alcohol, few policies have been implemented to minimize public consumption of alcohol or to mitigate associated risks [43]. To the extent that such policies may reflect and/or shape social perceptions of alcohol, their relative absence in Vietnam may indicate limited social awareness of alcohol’s addictive potential. Similarly, traditional perceptions of illicit drug use as a “social evil” tend to emphasize the role of personal choice, rather than addiction, in popular conceptions of methamphetamine use [41]. When experiencing symptoms of withdrawal following use of multiple substances, people holding such beliefs may tend to disproportionately attribute their withdrawal symptoms to the specific substance which they believe a priori to be addictive. Thus participants who used both heroin and methamphetamine or alcohol may have “blamed” their withdrawal symptoms largely upon heroin, and failed to fully consider the possibility that they were simultaneously withdrawing from methamphetamine or alcohol. In addition to these cultural factors, for nearly all participants initial methamphetamine use was relatively recent compared to much longer histories of opioid use. It is possible that these participants were relatively early in the course of their methamphetamine use and had not yet developed a dependence that would be perceptible to them in the context of their coexisting OUD.

Participants enrolled in the BRAVO MOUD treatment trial reported both methamphetamine and alcohol use as part of the social fabric of their daily experience. MOUD has been associated with increased rates of loneliness among people with OUD, and it is possible that use of methamphetamine and alcohol in social settings may be a strategy for coping with loneliness [45]. Alcohol has a long-established place in the culture and social life of Vietnam and is thought to have traditionally served to smooth-over intergroup tensions, a role for which social pressure (especially for men) to drink to excess is integral [46]. It is possible that alcohol’s traditional role in temporarily levelling otherwise rigid social hierarchies may serve to help PWID overcome stigma and maintain social connections with people who do not use drugs. An additional possibility is that a deeply ingrained social dynamic surrounding alcohol consumption, whereby individual members of a group are pressured to engage in binge drinking, may have been transferred to newly arrived psychoactive substances such as methamphetamine. The importance of methamphetamine and alcohol use in social settings in the current study also suggests that interventions addressing polysubstance use might target family and friend social networks in Vietnam. Such interventions are particularly needed for alcohol, which is an essential aspect of social and business life for many people in Vietnam. 

Our study identified complex interactions between methamphetamine, alcohol, and heroin use which are consistent with results seen elsewhere, as well as with proposed underlying mechanisms. For example, our study findings in Vietnam are similar to qualitative data from people who use drugs in rural communities in the United States, who believed methamphetamine could decrease heroin cravings and overdose risk, as well as alleviate opioid withdrawal symptoms [30]. In China, moreover, people who previously used heroin reported switching to methamphetamine with the goal of transitioning to a drug with lower perceived addiction potential [47]. Methamphetamine use is hypothesized to alleviate physiological symptoms of opioid withdrawal through activation of different dopamine reward pathways than those inhibited by MOUD [10, 48], suggesting a potential neurobiological basis for the perception that methamphetamine might be helpful for quitting heroin. Multiple genetic risk loci have been associated with heroin use, methamphetamine use, and heavy alcohol use, yet little is known regarding their interaction and behavioral correlates [49]. Studies demonstrating decreased injection frequency among PWID with increased alcohol consumption suggest that a relationship may exist between heroin and alcohol use [40]. A similar causal hypothesis between heroin and methamphetamine use may also exist.

Our study should be interpreted in light of potential limitations. Though lack of generalizability is not a threat to validity in qualitative research, study participants were people with HIV participating in a trial of two MOUD treatment strategies in urban Vietnam HIV clinics. Exclusion of people with significantly elevated liver enzymes from the main BRAVO trial may have limited the voice of people with more serious alcohol use. Still, our findings inform the relationship between methamphetamine, alcohol, and heroin use in the context of continued growth in methamphetamine prevalence and ongoing HIV transmission associated with injection drug use in Vietnam and throughout Southeast Asia and the U.S. [42].

## Conclusions

Further work is required to better elucidate the relationship between methamphetamine and alcohol in people who use opioids in both behavioral and basic science domains. A crucial first step is for OUD treatment trials to include measures of polysubstance use and analytic plans that assess relationships between complex use patterns and study outcomes. As the population of people receiving MOUD increases in Vietnam and globally, a better understanding of such relationships will be necessary for clinicians and policymakers to develop and implement effective interventions to treat patients with polysubstance use.

## Data Availability

The qualitative datasets generated and/or analysed during the current study are not publicly available to ensure subject privacy, but are available from the corresponding author on reasonable request.

## Abbreviations

AIDS: Acquired Immunodeficiency Syndrome
ART: Antiretroviral Therapy
ATS: Amphetamine-type Stimulant
HIV: Human Immunodeficiency Virus
IDU: Injection Drug Use
MOUD: Medication for Opioid Use Disorder
OUD: Opioid Use Disorder
PWID: People Who Inject Drugs
